# Effect of women’s age on embryo morphology, cleavage rate and competence—A multicenter cohort study

**DOI:** 10.1371/journal.pone.0172456

**Published:** 2017-04-19

**Authors:** Marie Louise Grøndahl, Sofie Lindgren Christiansen, Ulrik Schiøler Kesmodel, Inge Errebo Agerholm, Josephine Gabriela Lemmen, Peter Lundstrøm, Jeanette Bogstad, Morten Raaschou-Jensen, Steen Ladelund

**Affiliations:** 1 Herlev Hospital, Fertility Clinic, Copenhagen University Hospital, Herlev, Denmark; 2 Aarhus University Hospital, Fertility Clinic, Aarhus, Denmark; 3 Brædstrup Fertility Clinic, Horsens, Denmark; 4 Rigshospitalet, Fertility Clinic, Copenhagen University Hospital, Copenhagen, Denmark; 5 Ballerup IVF, Copenhagen, Denmark; 6 Hvidovre Hospital, Fertility Clinic, Copenhagen University Hospital, Hvidovre, Denmark; 7 CellCura Solutions A/S, Copenhagen, Denmark; 8 Hvidovre Hospital, Statistics, Clinical Research Center, Copenhagen University Hospital, Hvidovre, Denmark; Hull York Medical School, UNITED KINGDOM

## Abstract

This multicenter cohort study on embryo assessment and outcome data from 11,744 IVF/ICSI cycles with 104,830 oocytes and 42,074 embryos, presents the effect of women’s age on oocyte, zygote, embryo morphology and cleavage parameters, as well as cycle outcome measures corrected for confounding factors as center, partner’s age and referral diagnosis. Cycle outcome data confirmed the well-known effect of women’s age. Oocyte nuclear maturation and proportion of 2 pro-nuclear (2PN) zygotes were not affected by age, while a significant increase in 3PN zygotes was observed in both IVF and ICSI (p<0.0001) with increasing age. Maternal age had no effect on cleavage parameters or on the morphology of the embryo day 2 post insemination. Interestingly, initial hCG value after single embryo transfer followed by ongoing pregnancy was increased with age in both IVF (p = 0.007) and ICSI (p = 0.001) cycles. For the first time, we show that a woman’s age does impose a significant footprint on early embryo morphological development (3PN). In addition, the developmentally competent embryos were associated with increased initial hCG values as the age of the women increased. Further studies are needed to elucidate, if this increase in initial hCG value with advancing maternal age is connected to the embryo or the uterus.

## Introduction

It is well-recognized that the fertility potential of women decreases with increasing age. The decline in female reproductive capacity with increasing age has two main causes: Gradual depletion of oocytes from the ovary, and a decrease in oocyte quality [1].

A fully developmentally competent oocyte is an oocyte that through oogenesis achieves the ability to resume meiosis, become fertilized with a single spermatozoa, de-condense the sperm head, create two pronuclei (PN), pass cleavage stages, undergo maternal-to-embryonic transition, reach the blastocyst stage, hatch, and establish a pregnancy that results in the birth of a healthy baby [2]. That the decrease in the oocyte developmental competence contributes to lower fecundity with increasing age has been shown by achieving comparable pregnancy rates between young women undergoing *in vitro* fertilization (IVF) treatment with their own oocytes and older women (>40 years) in IVF treatment with oocytes donated by the younger women [3].

The aneuploidy rates in both oocytes and *in vitro* produced embryos increase with increasing female age [4–6]. In a recent study using array comparative genomic hybridization of polar bodies, the aneuploidy rate increased from 47% among women aged 27–37 years to 78% among those aged 38–47 years [6].

We have previously shown that there is a significant effect of age on the expression of genes involved in cell cycle regulation (e.g. microtubule-based processes, chromatin assembly, M-phase of the meiosis) in oocytes in metaphase II (MII) from women undergoing IVF or intra-cytoplasmic sperm injection (ICSI) treatment [7]. These findings supported that meiosis and cell cycle regulation mechanisms may be affected by increasing age of the woman.

In IVF and ICSI treatment, the oocytes, zygotes, and embryos are evaluated by cleavage rate and morphological parameters reported to correlate with favorable outcome, following the ALPHA and ESHRE consensus parameters [8]. It has previously been found that a summed score for cleavage stage embryo quality is not affected by women’s age [9,10], but it is not known if age influences the individual parameters and hence the more detailed cleavage events.

The aim of this study was to investigate whether individual oocyte, zygote, and embryo morphology and cleavage parameters of the developing embryo, as well as outcome measures are influenced by the age of the woman undergoing IVF or ICSI treatment.

## Materials and methods

### Design

Multicenter, historical cohort study.

### Data and patients

The data was collected from 5 Danish fertility clinics’ (4 public and 1 private clinic) databases (FertiClient, CellCura Solutions A/S, Copenhagen, Denmark). In the dataset received for analysis from the clinics' databases, subjects could not be identified, neither directly nor through identifiers linked to the subjects and, therefore, no institutional review board or ethics committee approval was needed according to Danish law.

All IVF and ICSI cycles following controlled ovarian stimulation (COS), except cycles with donor gametes or pre-implantation genetic diagnostic, performed during 2007–2010, were included. The regimens for COS included standard long agonist and short antagonist protocols with either recombinant FSH or highly purified menotrophin. Ovulation was induced by hCG or GnRH agonist administration when leading 2 follicles reached 17 mm, and oocyte pick up was performed 34–36 hours later. All five clinics used the same static time frames for assessing the various oocyte and embryo evaluation parameters, even though ‘early cleavage’ was only used in 2 centers (representing 37% of the cycles), and only one center used routine registration of multinuclear blastomeres (representing 20% of the cycles). All centers routinely transferred 1 or 2 embryos on day 2. Within centers, the *in vitro* procedures as well as the luteal phase support with progesterone and the method for hCG analysis were identical throughout the age groups. Potential influence of variation between centers regarding COS regimens and *in vitro* procedures on data is addressed in the statistical analysis model by stratification for center.

#### IVF and ICSI

The methods of insemination include differences in handling and induce differences in timing of cleavages post insemination and hence difference in timing for assessing the cleavage events has been recommended [11]. However, the routine embryo assessments in the 5 clinics did not include differential timing in regard to insemination technique and, therefore, we chose to split the embryo assessment data into IVF and ICSI.

In ICSI cycles, only MII oocytes were inseminated, while the immature oocytes were discarded.

#### Parameters

The data collected included the following parameters: Woman’s age, partner’s age, referral diagnosis, number of cycles, cycle type (IVF; ICSI), number of oocytes (number of cumulus-oocyte-complexes at oocyte retrieval 34–36 hours after ovulation trigger), number of MII oocytes (ICSI only: 38–40 hours after ovulation trigger), fertilization (0 PN; 1PN; 2PN; ≥3PN: 18–20 hours after insemination), early cleavage (PN number; cleavage: 24–26 hours after insemination), number of blastomeres (42–44 hours after insemination), fragmentation (0%; >0–10%; >10–25%; > 25%), blastomere size (even; uneven: data from 2- and 4-cell embryos only: 42–44 hours after insemination), and presence of multinuclearity (embryos with blastomere(s) with more than 1 nucleus or micronuclei, which are smaller nuclear structures (see Luzhna et al 2013(ref. 30)): 42–44 hours after insemination) in one or more blastomeres. Furthermore, number of embryos transferred on day 2 (1; 2), hCG in blood plasma (initial hCG value) (IU/L; positive (>25IU/L); negative (<25IU/L): 14 days after transfer), and number of gestational sacs and fetal hearts (0; 1; 2: week 7–8 of gestation) were extracted from the databases.

Primary endpoints are MII rate (ICSI) (number of MII / number of oocytes), fertilization rates (number of 2PN zygotes / number of oocytes inseminated; number of 1PN zygotes / number of oocytes inseminated; number of ≥3PN zygotes / number of oocytes inseminated), cleavage rate (number of 2PN zygotes cleaved / number of 2PN zygotes), early cleavage rates (number of 2PN zygotes early cleaved / number of 2PN zygotes; number of 0PN zygotes early cleaved / number of 0PN zygotes (0PNs were followed to ensure observations of zygotes where PN had faded before the fertility check)), 2-cell rate (number of 2PN zygotes being 2-cells at day 2 / number of 2PN zygotes), 4-cell rate (number of 2PN zygotes being 4-cells at day 2 / number of 2PN zygotes), rate of 4-cells with moderate to high (>10%) fragmentation, 4-cells with uneven sized blastomeres at day 2, and rate of 4-cells with one or more multinuclear blastomeres.

Secondary endpoints are number of oocytes, cycle outcomes as embryo transfer (ET) rate (number of embryo transfers / number of oocyte retrievals), single embryo transfer (SET) rate (number of ETs with a single embryo / total number of ETs), positive hCG rate per embryo transfer, implantation rate (number of gestational sacs / number of embryos transferred), clinical intra uterine pregnancy rate (number of pregnancies with gestational sac / ET), and ongoing pregnancy rate (number of pregnancies with fetal heart beat / ET). Initial value in hCG in cycles with single embryo transfer resulting in ongoing pregnancy was evaluated in a post-hoc analysis.

For the data set see S1 Table.

### Statistical methods

Data are presented as counts and percentages or as means, where appropriate. Parameters from different levels of nesting were analyzed for age differences. Parameters regarding single oocytes/embryos were nested within cycles, which in turn were nested within women. Parameters describing cycles, e.g. number of oocytes extracted, were nested within woman alone. Finally, some parameters show no nesting at all, such as partner’s age at first cycle. The parameters under evaluation represented counts and binary data. We, therefore, chose to use generalized linear models with random effects according to nesting levels, thus allowing for the correlated nature of the data, to model the outcomes, taking into account potentially confounding variables, including treatment center, partner’s age (categorical: <30, 30–50, >50 years), and referral diagnosis. This comprises mixed logistic regression for binary data reporting Adjusted Odds Ratios (OR), and mixed Poisson regression for count data reporting intensity rate ratios. R-2.15 [12] was used for data-management and statistical analysis. The lme4 package [13] was used for fitting the multivariate generalized mixed models. Age differences were analyzed by means of age groups, as well as applying a test for trend. Treatment centers were considered a fixed effect in all multivariate analyses. Total FSH dose was considered a potential intermediate variable and was therefore not adjusted for. To account for potential effect modification/interaction, analyses for main outcomes were stratified by center. Missing data was considered missing completely at random and incomplete cases thus removed from analysis. In one treatment center (representing 27% of the cycles), data on referral diagnosis were missing, and subsequently coded as “other”. P-values under 0.05 were considered statistically significant.

## Results

In total, 11,744 cycles (5650 IVF and 6094 ICSI) were included in the analysis representing 104,830 oocytes and 42,074 embryos.

### Partner’s age and infertility diagnosis

Partner’s mean age and referral diagnosis by female age groups are shown in Table 1. Both were significantly correlated to female age, and hence the subsequent analyses were adjusted for these confounding variables.

**Table 1:** Partner’s age and referral diagnosis in relation to the age of the women undergoing IVF or ICSI.

	Female age group (years)						
	18–24	25–29	30–35	36–40	41–46	p-value	p-value, *Test for trend*^*#*^
**Partner’s age (mean years)**	28.3	32.1	35.1	38.7	42.1	<0.0001	<0.0001
**Tubal factor**	16 (5.7%)	107 (4.4%)	316 (7.0%)	455 (11.9%)	36 (5.1%)	<0.0001	0.16
**Male factor**	123 (43.5%)	921 (38.2%)	1571 (34.7%)	1099 (28.7%)	192 (27.2%)	<0.0001	<0.0001
**Anovulation**	29 (9.2%)	192 (8.0%)	276 (6.1%)	167 (4.4%)	16 (2.3%)	<0.0001	<0.0001
**Endometriosis**	3 (1.1%)	77 (3.2%)	135 (3.0%)	88 (2.3%)	3 (0.4%)	<0.0001	<0.0001
**Other**	115 (40.6%)	1112 (46.2%)	2225 (49.2%)	2015 (52.7%)	458 (65.0%)	<0.0001	0.00012

p<0.05 is considered significant.

^#^Modeling age groups in a linear fashion.

#### Cycle outcome

Table 2 shows the outcome of IVF and ICSI cycle in regard to female age. The number of cycles per woman increased with increasing age. Furthermore, the number of oocytes retrieved decreased with increasing age. For both IVF and ICSI cycles, no significant age dependency was found in the chance to have embryo transfer after oocyte retrieval, while the rate of single embryo transfers significantly decreased with increasing age (p<0.0001). A significant decrease in cycle clinical outcome parameters (positive hCG, clinical intrauterine pregnancy, as well as ongoing pregnancy), were present with increasing age of the woman (p<0.0001).

**Table 2:** The effect of women’s age on treatment practice and clinical outcome measures after IVF and ICSI.

		Female age group (years)						
Measurements	Cycle	18–24	25–29	30–34	35–39	40–46	p-value	p-value, *Test for trend*^*#*^
**No. of cycles per age group**		283 (2.4%)	2409 (20.5%)	4523 (38.5%)	3824 (32.6%)	705 (6%)		
**No. of cycles per woman (mean)**		1.66	1.74	1.83	1.94	2.07	<0.0001	<0.0001
**No. of oocytes retrieved per cycle (mean)**		9.63	9.82	9.40	8.03	7.41	<0.001	<0.0001
**Embryo Transfer (%)**	IVF	86.1	90.5	89.9	90.4	90.2	>0.1	0.86
	ICSI	92.6	91.8	90.3	86.0	84.5	>0.1	0.37
**Single embryo transfer rate (%)**	IVF	54.5	52.3	43.8	33.2	27.1	<0.0001	<0.0001
	ICSI	54.5	48.2	40.6	36.0	31.2	<0.0001	<0.0001
**Positve hCG (%)**	IVF	38.9	40.0	38.6	32.7	17.4	<0.0001	<0.0001
	ICSI	41.7	38.9	35.3	31.2	16.5	<0.0001	<0.0001
**Clinical intrauterine pregnancy (%)**	IVF	30.6	33.6	31.1	25.9	12.3	<0.0001	<0.0001
	ICSI	37.7	32.8	28.9	24.9	13.5	<0.0001	<0.0001
**Ongoing pregnancy (%)**	IVF	29.6	31.1	28.9	22.9	9.6	<0.0001	<0.0001
	ICSI	36.0	30.1	26.6	22.0	10.1	<0.0001	<0.0001
**Implantation rate (%)**	IVF	24.66	25.31	23.33	17.79	8.51	<0.0001	<0.0001
	ICSI	29.03	24.43	21.59	18.47	9.85	<0.0001	<0.0001

p<0.05 is considered significant.

^#^Modeling age groups in a linear fashion.

**Fig 1:**
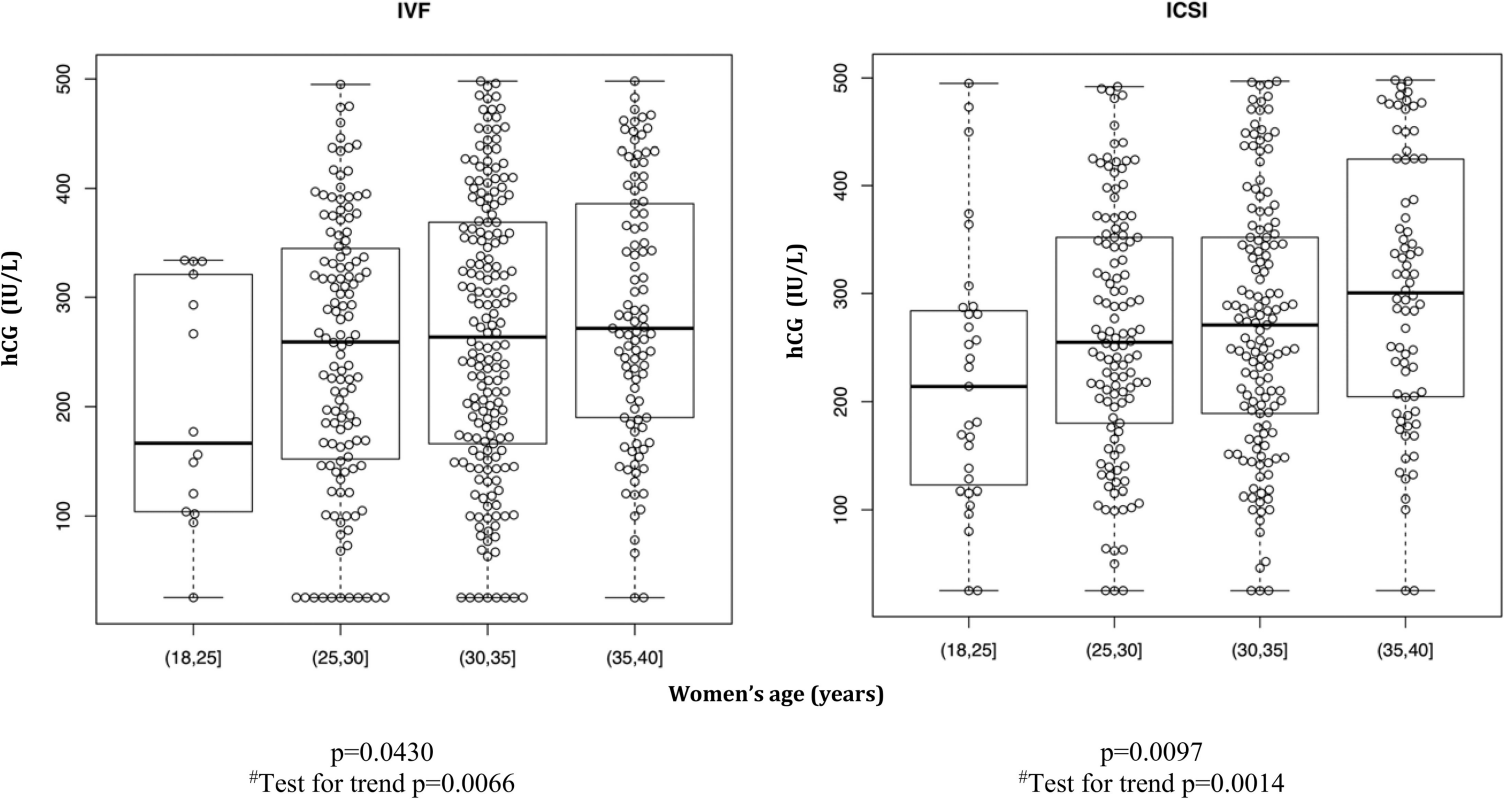
The initial rise* in hCG after single embryo transfer in IVF and ICSI treatments resulting in ongoing clinical pregnancy with fetal heart beat (week 7–8) increases significantly with increasing women’s age. *hCG in blood plasma 14 days after day-2 transfer. Number of observations: IVF: 429; ICSI: 374. ^#^ Modeling age groups in a linear fashion.

The initial value in hCG in IVF and ICSI cycles with single embryo transfer resulting in ongoing pregnancy (ICSI: 374; IVF: 429) is shown in Fig 1. In both IVF and ICSI cycles, the initial value in hCG increased with increasing age of the woman. This analysis was not stratified for referral diagnosis due to limited size of the data set.

### Morphology and cleavage rate parameters

Table 3 shows the oocyte, zygote, and embryo evaluations parameters.

**Table 3:** Effect of women’s age on key morphology and cleavage evaluation parameters in oocyte, zygote and embryo in 5,650 IVF and 6,094 ICSI cycles.

			Female age group (years)						
Evaluation-parameter	Cycle		18–24	25–29	30–34	35–39	40–46	p-value	p-value, *Test for trend*^#^
**MII rate**^**a**^	ICSI	%	79.4	80.0	79.1	79.5	79.2		
		OR	1	0.96 (0.74–1.24)	0.95 (0.73–1.24)	0.98 (0.74–1.28)	0.82 (0.57–1.17)	0.73	0.69
**Fertilization-rate, *2PN***^***b***^	IVF	%	55.6	56.4	56.1	58.5	53.8		
		OR	1	0.83 (0.63–1.08)	0.63 (0.63–1.08)	0.93 (0.71–1.22)	0.79 (0.58–1.08)	0.02	0.22
	ICSI	%	53.1	58.8	58.1	59.3	58.2		
		OR	1	1.32 (1.07–1.61)	1.29 (1.05–1.58)	1.31 (1.06–1.62)	0.99 (0.75–1.31)	0.007	0.90
**Fertilization-rate, *1PN***^***b***^	IVF*	%	3.9	4.1	3.4	3.4	3.3		
		OR	NA	NA	NA	NA	NA	NA	NA
	ICSI	%	4.9	3.3	3.1	3.2	2.6		
		OR	1	0.74 (0.64–0.86)	0.75 (0.64–0.88)	0.70 (0.59–0.82)	0.90 (0.72–1.12)	<0.0001	0.08
**Fertilization-rate, *≥3PN***^***b***^	IVF	%	5.15	4.4	4.7	4.9	6.8		
		OR	1	0.92 (0.64–1.32)	1.15 (0.80–1.65)	1.24 (0.86–1.78)	1.71 (1.13–2.57)	<0.0001	<0.0001
	ICSI	%	2.4	1.7	1.7	2.8	3.4		
		OR	1	0.80 (0.49–1.31)	0.97 (0.59–1.60)	1.31 (0.79–2.18)	1.69 (0.91–3.14)	0.0003	<0.0001
Earlycleavage in 2PN zygotes^c^**	IVF	%	25.6	24.6	23.7	22.8	27.3		
		OR	1	0.88 (0.42–1.83)	0.88 (0.43–1.84)	0.88(0.42–1.85)	0.90 (0.95–2.29)	1.00	0.92
	ICSI	%	33.2	29.7	26.6	22.5	24.7		
		OR	1	0.90 (0.48–1.66)	0.81 (0.43–1.54)	0.62 (0.32–1.21)	0.78 (0.25–2.40)	0.38	0.06
**Cleavage/0PN^d^**	IVF	%	12.7	14.4	11.8	12.7	13.5		
		OR	1	0.64 (0.12–3.44)	0.46 (0.08–2.50)	0.49 (0.09–2.76)	0.69 (0.09–5.11)	0.15	0.18
	ICSI	%	14.4	13.3	11.7	9.6	12.3		
		OR	1	0.74 (0.13–4.22)	0.55 (0.09–3.36)	1.08 (0.17–7.02)	2.17 (0.19–24.8)	1.00	0.02
**2-cell rate/2PN**^**d**^	IVF	%	10.5	16.4	17.7	20.0	18.7		
		OR	1	1.55 (0.95–2.54)	1.71 (1.04–2.79)	1.88 (1.14–3.08)	1.45 (0.82–2.55)	0.35	0.07
	ICSI	%	19.0	18.4	20.7	20.2	22.8		
		OR	1	1.03 (0.69–1.54)	1.25 (0.83–1.88)	1.09 (0.71–1.66)	1.21 (0.71–2.05)	0.22	0.58
**4-cell rate/2PN**^**d**^	IVF	%	42.9	44.7	44.6	41.7	41.8		
		OR	1	0.82 (0.55–1.23)	0.79 (0.53–1.18)	0.77 (0.51–1.16)	0.60 (0.37–0.96)	0.24	0.07
	ICSI	%	46.3	43.6	41.9	41.1	41.2		
		OR	1	0.91 (0.66–1.26)	0.82 (0.59–1.15)	0.89 (0.63–1.25)	0.73 (0.47–1.15)	0.44	0.40
**Fragmentation>10%, 4cell**^**d**^	IVF	%	26.8	32.6	31.5	34.1	34.1		
		OR	1	1.17 (0.67–2.05)	1.14 (0.65–2.01)	1.42 (0.80–2.53)	1.33 (0.68–2.60)	0.21	0.06
	ICSI	%	35.5	34.4	34.0	34.7	32.7		
		OR	1	0.90 (0.58–1.40)	1.00 (0.63–1.57)	1.06 (0.66–1.71)	1.05 (0.55–1.99)	0.76	0.25
**Blastomere size Unequal, 4-cell**^**d**^	IVF	%	35.2	33.5	32.1	35.9	27.8		
		OR	1	0.85 (0.59–1.21)	0.75 (0.52–1.07)	0.85 (0.59–1.22)	0.65 (0.41–1.02)	0.07	0.51
	ICSI	%	34.8	38.4	37.9	38.7	27.5		
		OR	1	1.03 (0.74–1.44)	1.02 (0.72–1.44)	0.93 (0.65–1.33)	0.77 (0.46–1.27)	0.48	0.14
Multinuclearity4-cell^d^***	IVF	%	9.2	7.1	7.7	8.3	6.9		
		OR	1	0.70 (0.28–1.73)	0.68 (0.28–1.68)	0.80 (0.32–1.99)	0.58 (0.14–2.35)	0.81	0.88
	ICSI	%	10.8	11.1	9.4	10.9	0		
			1	0.76 (0.31–1.87)	0.58 (0.22–1.50)	0.70 (0.26–1.85)	NA	0.51	0.58

OR: Adjusted Odds ratio (adjusted for treatment center, partner’s age and referral diagnosis). p<0.05 is considered significant.

^#^Modeling age groups in a linear fashion.

^a^Day 0, 38–40 hours after ovulation trigger.

^b^Day 1, 18–20 hours after insemination.

^c^Day 1, 24–26 hours after insemination.

^d^Day 2, 42–44 hours after insemination.

*Multivariate analysis was not possible due to lack of variation.

**Early cleavage was evaluated in two centers only (number of embryos assessed: IVF: 14,214; ICSI: 10,241).

***Blastomere multinuclearity was evaluated in one center only (number of embryos assessed: IVF: 6,623; ICSI: 4,447).

The maturity of the oocytes, evaluated at the time of ICSI, was not influenced by the age showing a constant MII rate around 80% throughout the dataset.

The analysis to reveal a potential age dependent trend in 2PN fertilization rate did not show a significant effect in either IVF or ICSI cycles (Table 3). However, the p-value indicated a difference in 2PN rates between the age groups. In ICSI cycles, the youngest age (18–24 years) group (OR = 1) showed lower rate as compared to the groups 25–29, 30–34 and 35–39 years (all OR significantly >1). In IVF cycles, the youngest age group showed a non-significant difference in rate compared to all older age groups. As the scope of the study was to address a general effect of age, further pairwise comparisons between individual groups were not performed.

In both IVF and ICSI, the rates of 3PN zygotes were significantly increased with age (p-value test for trend, p<0.0001). In ICSI, the prevalence of 1PN zygotes decreased with increasing age, although the test for trend did not reach statistical significance (p-value test for trend, p = 0.08). For the 1PN rate in IVF cycles, no multivariate analysis was available due to lack of variation.

The rates of unequally sized blastomeres in 4-cell embryos were unaltered by female age representing about 35% of the 4-cell embryos.

No significant differences in fragmentation were present at the 4-cell stage. However, a systematic tendency towards increased fragmentation at the 4-cell stage in IVF embryos was observed, although not significant (p-value test for trend, p = 0.06).

Multinuclear blastomeres were found in 40–45% of 2-cell and 3-cell stage embryos (data not shown) in both IVF and ICSI, while only 7–11% of 4-cell stage embryos after IVF and ICSI contained multinuclear blastomeres. The rate of multinuclear embryos was not influenced by the age of the woman (data for 2- and 3-cells are not shown, for 4-cells please see Table 3).

The cleavage rate (approximately 90%) per 2PN-zygote was not influenced by age (IVF: test for trend p = 0.16; ICSI: test for trend p = 0.33, these data are not shown in Table 3). For ICSI zygotes, a reduced rate in early cleavage was present, albeit insignificant (test for trend p = 0.06) with increasing age, while no trend was present for IVF (test for trend p = 0.92). Regarding the rate of 0PN zygotes, cleaving was also decreased by increasing female age after ICSI (test for trend p = 0.02), while no age effect was found for this rate in 0PN zygotes after IVF. For ICSI, no trend in age-induced 2- or 4-cell rate (day 2) was present, while a statistically insignificant tendency towards an increase in 2-cell rate (test for trend p = 0.07), and reduction in 4-cell rate (test for trend p = 0.07) were present in the IVF embryos.

The analysis stratified by center (number of oocytes, 3PN prevalence and initial hCG value) showed homogeneous results for all strata (data not shown).

Re-analysis of the data without the youngest age group was performed to ensure that the observed effect of age remained if the youngest age group was excluded. Test for trends in this re-analysis confirmed the age effect and level of significance in all parameters in Tables 2 and 3. Likewise, for the initial hCG value after SET of a competent embryo the trend was confirmed for IVF (p = 0.03) and ICSI (p = 0.06).

## Discussion

This is the first report on the influence of age on the morphology of the early human embryo. The increase in prevalence of 3PN zygotes is observed in both IVF and ICSI with increasing age, which may suggest that the background for the increase in 3PN is a compromised ability to extrude the second polar body, rather than compromised polyspermic block. Considering the 3PN zygote as the ultimate incompetence in 2nd meiosis, the increase in the 3PN prevalence with increasing age may reflect the underlying general insufficient function of the meiotic metaphase spindles and affected expression of genes related to M-phase of meiotic cell cycle in the aged mammalian [14] and human oocytes [7], respectively. The age response in 3PN tends to be u-shaped as do several of the outcome measures with the youngest group of women (18–25 years) showing compromised performance. Recent reviews report the same u-shaped tendency in aneuploidy rate in human assisted reproductive technology blastocysts [15] and clinical outcome [16] additionally indicating that the 3PN prevalence may relate to compromised meiotic competence in the cohort of oocytes. However, it cannot be excluded that age also affects the mechanism of the polyspermic block, as we did not have data on the number of polar bodies 18–20 hours post insemination.

Our data on cycle outcome confirmed the well-documented age-induced decline in embryo competence, while the significantly higher initial value of hCG in both IVF and ICSI singleton pregnancies following SET with increasing woman’s age is new. If the correlation between age and a higher level of hCG relies on altered trophectoderm cells or endometrial conditions needs to be elucidated. Both ploidy and age-related unique changes in human blastocysts microRNA profile have been reported recently [17]. The present hCG data were not adjusted for differences in stimulatory regimens used in the cycles, and hence it cannot be excluded that the observed effect is due to differences in COS which have been shown to influence the gene expression of the endometrium [18,19].

The constant rate (80%) of nuclear matured oocytes indicates that the age of the woman does not impair the ovulation’s trigger-induced signal to resume meiosis. This is in contrast to a recent publication reporting a significantly higher rate of immature oocytes after controlled ovarian stimulation in women over forty years-of-age as compared to younger women [20]; however, their finding was based on a limited (< 200 cycles) number of observations with no adjustment for referral diagnosis.

The fertilization rate (2PN) was not influenced by the increasing age of the women in IVF or ICSI confirming previous findings [9,10]. However, minor variation between age groups were seen and in ICSI cycles, the youngest age group did show a lower fertilization rate as compared to the other groups. The competence to progress from 2PN to cleavage, showed no correlation with age, while the timing of the first cleavage (early cleavage) in ICSI 2PN zygotes, however, showed a tendency to be postponed by increasing age of the woman; from 40% of 2PN zygotes being early cleaved in the youngest age group to 25% in the eldest. IVF embryos had a tendency to increased length between cell cleavages with increasing age of the woman, having increased and decreased proportion of 2-cell and 4-cell embryos, respectively, at day 2. Different cleavage parameters being affected by age in IVF and ICSI embryos probably reflects the difference in timing of the assessment relative to actual fertilization time, rather than difference in the development of IVF and ICSI zygotes. The post insemination difference in cleavage timing after IVF and ICSI is well-recognized [11]; thus, splitting of the present embryo evaluation data set into IVF and ICSI probably provided us with more time differentiated picture of the cleavage events.

Recent studies addressing the effect of age on cleavage stage development showed no association between age and a summed score of the embryo at day 2 and 3 [9,10]. Neither of these studies assessed early cleavage, nor did they analyze prevalence of the various cell stages, which may explain the discrepancies.

The age-induced impairment of embryo competence is strongly associated with aneuploidy in human embryos, which originates predominantly from mis-segregation in meiosis [5,6], and to a minor degree from early mitotic errors [6]. Chavez and colleges (2012) reported increased duration of the first cytokinesis in human frozen-thawed zygotes, as well as an increase in length of the second cell cycle in aneuploid as compared to euploid embryos [21]. A delayed early cleavage division has also been confirmed to relate to aneuploidy [22], while a recent time-lapse study failed to associate ploidy to cleavage stages parameters [23]. The present data showing a tendency towards increased time between cell cleavages with increasing maternal age further supports that the cleavage rate may be associated with chromosomal status of the embryo. If the delay in timing of events starts already at the 1st meiotic division is not known. However, no major delay seems to be present, since the MII rate was independent of age. The 2nd meiotic division may be influenced by age, since a recent publication comparing two age groups reports increased time to 2nd polar body extrusion after ICSI in women aged ≥38 years as compared to younger women [24]. This is in line with the effects of age on initial cleavages in the present study.

Future time-lapse recordings of oocytes following ICSI will allow detailed registration of timing of second polar body extrusion over PN appearance, PN fading, and first cleavage, and reveal if the age-induced delay is established during the meiosis and/or the preparation for the first mitosis. An increase in anaphase lag in oocyte meiosis with increasing age has already been shown in mice [25,26].

Previous reports have shown no age [9,10] or aneuploidy [6] dependent effect on summed morphology score that included blastomere size (even; uneven) without including cell stage specificity. The present data take the morphology analysis further, and show no association between maternal age and the blastomere size regularity in 2- and 4-cell embryos, indicating normal formation of cleavage furrow and cytokinesis during the first cleavages. Time-lapse studies have elucidated that fragments are generated dynamically during cytokinesis [27,28], and while failing to associate with euploidy as single parameter, dynamic fragmentation is suggested to correlate to euploidy [21]. In our data, no age effect was found in single time point fragmentation degree in neither 2-cell (IVF and ICSI), nor 4-cell (ICSI) embryos. However, we observed a tendency in increased fragmentation rate in the 4-cells after IVF by increasing age supporting previous findings in IVF embryos day 2 [29]. We speculate that this may reflect an age-related increase in dynamic fragmentation related to the second cleavage visible in 4-cell embryos (IVF) in our data set, since the assessment time being closer to the cleavage event than in the 2-cells, and the 4-cells after ICSI.

The presence of more than one nucleus and/or micronuclei is correlated to disrupted cell cycle control and genomic instability [30], and in human cleavage stage embryos the frequency of chromosomally aberrant blastomeres is significantly higher in embryos containing multinuclear blastomeres as compared to mononucleated embryos [31]. High rates (40–50%) of multinuclear blastomeres in 2-cell and 3-cell embryos and lower (10%) in 4-cell embryos at day 2 were observed. On neither cell stages, there was an association with age in presence of blastomeres with more than 1 nucleus, suggesting that multinuclearity, observed at a single time point, is unrelated to the age-induced chromosomal instability and impairment of developmental competence.

Our study has several strengths: It is a large multicenter cohort study with prospectively collected data registered in an everyday setting, and hence reflects daily practice. Furthermore, we performed adjustment for multiple potential confounders.

In contrast to a general low rate of missing data, 27% of ‘referral diagnosis’ data were missing due to missing data from one center potentially imposing selection bias, which is a potential weakness of the study. However, the distribution of referral diagnoses is unlikely to differ from that of the other clinics, considering the fairly homogeneous population in Denmark.

In conclusion, while the MII rate and 2PN rate were unaffected, age does induce a significant effect on the prevalence of 3PN zygotes in both IVF and ICSI with increasing age, which may represent a compromised meiotic spindle function. The present large-scale study showed that in terms of individual static embryo evaluation parameters of embryo cleavage rate and morphology, the age-associated changes are primarily subtle. There was a tendency towards increased length between the early cell cleavages in both IVF and ICSI, and an increased fragmentation (in IVF only), which is in line with affected cell cycle and the age-related dominating symptom: aneuploidy. Our data on final outcome measures, such as implantation rate and ongoing pregnancy, confirmed the significant effect of age, however, the effect of women´s age on the initial hCG value after SET of competent embryos is new. Further studies are needed to elucidate, if the increase in initial hCG value with advancing maternal age is connected to the embryo or the uterus.

## Supporting information

S1 Table Data Groendahl et al. csv. (XLS)
